# Antibiotic Loaded Phytosomes as a Way to Develop Innovative Lipid Formulations of Polyene Macrolides

**DOI:** 10.3390/pharmaceutics16050665

**Published:** 2024-05-16

**Authors:** Svetlana S. Efimova, Olga S. Ostroumova

**Affiliations:** Laboratory of Membrane and Ion Channel Modeling, Institute of Cytology of Russian Academy of Sciences, Tikhoretsky Ave. 4, 194064 St. Petersburg, Russia; ostroumova@incras.ru

**Keywords:** amphotericin B, liposomes, lipid bilayers, membranes, sterols, phytosomes

## Abstract

Background: The threat of antibiotic resistance of fungal pathogens and the high toxicity of the most effective drugs, polyene macrolides, force us to look for new ways to develop innovative antifungal formulations. Objective: The aim of this study was to determine how the sterol, phospholipid, and flavonoid composition of liposomal forms of polyene antibiotics, and in particular, amphotericin B (AmB), affects their ability to increase the permeability of lipid bilayers that mimic the membranes of mammalian and fungal cells. Methods: To monitor the membrane permeability induced by various polyene-based lipid formulations, a calcein leakage assay and the electrophysiological technique based on planar lipid bilayers were used. Key results: The replacement of cholesterol with its biosynthetic precursor, 7-dehydrocholesterol, led to a decrease in the ability of AmB-loaded liposomes to permeabilize lipid bilayers mimicking mammalian cell membranes. The inclusion of plant flavonoid phloretin in AmB-loaded liposomes increased the ability of the formulation to disengage a fluorescent marker from lipid vesicles mimicking the membranes of target fungi. *I*–*V* characteristics of the fungal-like lipid bilayers treated with the AmB phytosomes were symmetric, demonstrating the functioning of double-length AmB pores and assuming a decrease in the antibiotic threshold concentration. Conclusions and Perspectives: The therapeutic window of polyene lipid formulations might be expanded by varying their sterol composition. Polyene-loaded phytosomes might be considered as the prototypes for innovative lipid antibiotic formulations.

## 1. Introduction

The growth of invasive mycoses caused by *Candida* spp. (candidiasis) and *Aspergillus* spp. (aspergillosis) has become a global problem in recent decades [1,2]. This group of infections is associated with a high mortality in cancer and immunocompromised patients. The rapid spread of drug resistance in fungi pathogens and the high toxicity of the most effective antibiotics requires a search for new antifungal agents and the development of innovative formulations of drugs.

Polyene macrolide antibiotics are the most efficient antifungal drugs currently used to treat invasive mycoses. Until now, amphotericin B (AmB) remains the main drug for the fight against invasive mycosis and is used in the treatment of leishmaniasis, invasive aspergillosis, candidiasis, and cryptococcal meningitis. Different authors agree that the antifungal effect of polyene macrolides and, in particular, AmB is determined by its membrane activity, and the key factor is the presence of sterols in the membranes of target cells. Despite many years of intensive research on the mechanisms of action of polyene macrolides, their interactions with lipid membranes remain poorly understood. The dominant concept is based on the formation of transmembrane pores by polyene macrolides in the membranes of fungal cells [3,4,5,6,7,8,9,10]. The alternative hypothesis (so-called sterol sponge model) is associated with the extraction of membrane sterols by polyenes to form extramembranous aggregates [11,12,13,14]. The specificity of polyene macrolide action on various cells is determined by the membrane sterol composition. It is important that mammalian and fungal cells differ in their sterol composition: the membranes of mammalian cells contain cholesterol (CHOL), while the fungal cell membranes are enriched with ergosterol (ERG). The affinity of polyene macrolides to ERG is higher than to CHOL, but the CHOL-containing mammalian cell membranes might be destroyed. This is the reason for high toxicity of antibiotics. It is under debate as to what is the key factor underlying polyene-sterol interactions: the stability of polyene-sterol complexes [15] or the differences in physicochemical properties of the lipid microdomains enriched with different sterols [16]. It is believed that AmB is capable of inducing two types of pores depending on the way it is added to lipid membranes. When AmB is introduced from one side of the membrane, it causes the formation of single-length cation-selective channels at antibiotic concentrations of one to two orders of magnitude higher than in the case of both-side addition of the antibiotic and the formation of double-length anion-selective channels [3,7,17]. Such a difference in threshold antibiotic concentrations can be the basis for the development of an approach for increasing the effectiveness of action of polyene macrolides by potentiating a transition from single-length to double-length polyene channels. Recently, using NMR and molecular dynamic simulation, the structure of AmB double- and single-length channels in ERG-containing membranes have been resolved, and alternative structures such as polyene- and sterol-enriched sponges have been shown to be extremely unlikely [18].

The toxicity, especially nephropathy [19], is a serious limitation to the use of polyene antibiotics in clinical practice [20,21]. Modern pharmaceutical approaches are aimed at reducing the toxicity of polyene macrolides. One way is to increase polyene water solubility by chemical modification [22,23,24,25,26,27]. Since the 1980s, lipid-associated forms of AmB have been also developed to reduce side effects: Abelcet^®^, Amphotec^®^, and Ambisome^®^ (liposomal AmB) [28,29,30,31,32]. However, controversy over the clinical efficacy, the tolerability, and the relatively high cost of treatment of the macrolide-based drug is still ongoing. Several attempts to create innovative lipid formulations of polyene macrolides have been described in the literature. The micellar composition of AmB was obtained upon the covalent attachment of CHOL molecules to the main chain of AmB and maleic acid [33]. Such micellar nanoparticles turned out to be water-soluble and stable in the presence of salts. Compared to a standard drug, Fungizone^®^ (AmB deoxycholate), this formulation showed equivalent antifungal activity, improved circulation, and less toxicity. The ionic amphiphilic preparation of AmB, called Kalsome™10, was also developed [34]. These liposomal structures are composed of an equimolar ratio of phosphatidylcholine and ERG with the addition of AmB and is characterized by less nephrotoxicity than Fungizone^®^. Iman and coauthors showed that the in vivo activity of DSHemsPC-AMB-Lip (AmB liposomal formulation enriched with synthetic lipid, 1,2-distigmasterylhemisuccinoyl-*sn*-glycero-3-phosphocholine) was comparable to that of Ambisome^®^ and was characterized by less cost in production [35]. A detailed study of the interactions among polyene antibiotics and their lipid-associated forms with model membranes should be carried out to identify the ways to further improve the pharmacological properties of polyene-based lipid formulations. 

Combining AmB with small natural molecules is another way to potentiate antibiotic efficacy [36,37,38,39,40]. We previously showed that a number of amphiphilic compounds of plant origin related to flavonoids, stilbenes, and alkaloids were able to enhance the pore-forming activity of pure AmB and its close analog, nystatin, in ERG-containing lipid bilayers by affecting polyene-sterol interactions or membrane elastic stress [6,7,41,42,43].

In this work, we assess the impact of the sterol and phospholipid composition of AmB liposome formulations on their ability to increase the permeability of lipid bilayers that mimic the membranes of mammalian and fungal cells. In the presented study, we also make an attempt to enhance the activity of AmB by including plant flavonoids in antibiotic-loaded liposomes.

## 2. Materials and Methods

### 2.1. Materials

**Figure 1:**
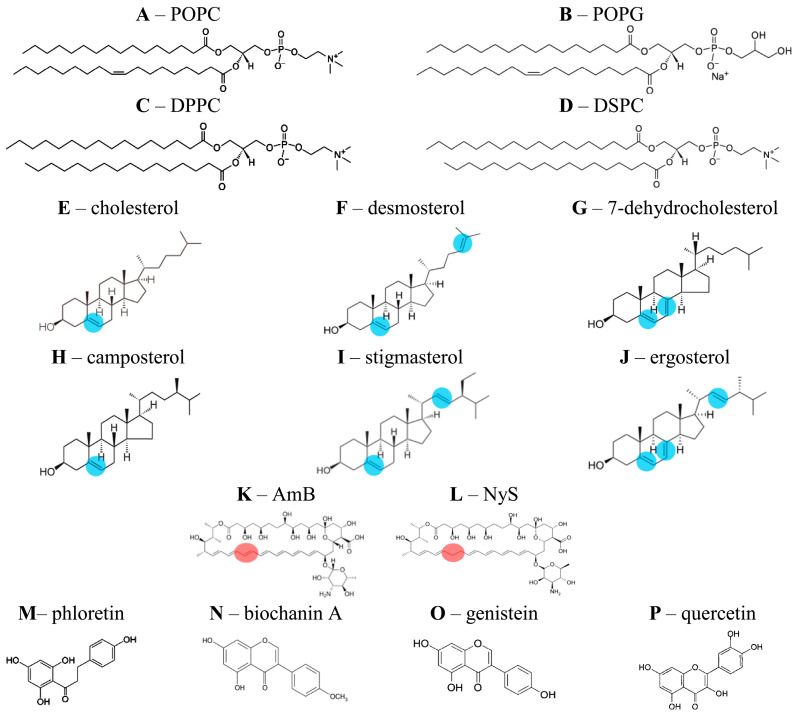
Chemical structures of phospholipids (**A**–**D**), sterols (**E**–**J**), antifungal polyene macrolides (**K**,**L**), and flavonoids (**M**–**P**) used in this study.

Synthetic 1-palmitoyl-2-oleoyl-*sn*-glycero-3-phosphocholine (POPC), 1-palmitoyl-2-oleoyl-*sn*-glycero-3-phospho-(1′-rac-glycerol) (POPG), 1,2-dipalmitoyl-*sn*-glycero-3-phosphocholine (DPPC), 1,2-distearoyl-*sn*-glycero-3-phosphocholine (DSPC), cholesterol (CHOL), stigmasterol (STIGM), 7-dehydrocholesterol (7DCHOL), desmosterol (DESM), and camposterol (CAMPO) were purchased from Avanti Polar Lipids (Avanti Polar Lipids, Inc., Alabaster, AL, USA). Amphotericin B (AmB), nystatin (NyS), ergosterol (ERG), phloretin, biochanin A, genistein, quercetin, NaCl, KCl, NaOH, KOH, HEPES, EDTA, dimethylsulfoxide (DMSO), triton X-100, calcein, sephadex G-50, ethanol, methanol, chloroform, and hexadecane were obtained from Sigma-Aldrich Company Ltd. (Gillingham, UK). Figure 1 presents the chemical structures of the phospholipids, sterols, antibiotics, and polyphenols used in this study.

### 2.2. The Production of the Polyene Lipid Formulations

To obtain the polyene-lipid formulations, the technique by [44] with modifications was used. A lipid mixture containing phospholipid (pure POPC or equimolar mixture of POPC and POPG, DPPC or DSPC) and sterol (CHOL, 7DCHOL, DESM, STIGM, CAMPO, or ERG) at a phospholipid:sterol molar ratio of 67:33 mol.% was suspended in a mixture of chloroform and methanol (67:33 vol.%). The total lipid concentration was equal to 275 µM. At this stage, antibiotics (AmB or NyS) and (or) flavonoids (phloretin, biochanin A, genistein, or quercetin) were added to some samples until there was an equimolar ratio of antibiotics, flavonoids, and lipids. The resulting solution was evaporated using a vacuum rotary evaporator (Hei-VAP Advantage HB/G3B ML, Heidolph, Germany) at 50 °C for 120 min. Further, the obtained film was hydrated by a buffer of 0.15 M KCl (10 mM HEPES, 1 mM EDTA, pH 7.4) and processed with an ultrasound for about 10 min. The list of the tested liposome formulations is presented in Table 1.

### 2.3. Antibiotic Concentration Assessement with Absorbance Spectroscopy

In order to build the absorbance spectra, the formulations containing AmB or NyS were scanned from 250 to 450 nm with a 1 nm step using a spectrofluorimeter, Fluorat-02-Panorama, produced by Lumex (Saint-Petersburg, Russia) at 25 °C. The AmB and Nys levels in samples were determined via optical density using the appropriate extinction coefficients in DMSO at 425 and 306 nm (related to the monomeric forms of polyenes), respectively. The control formulations without antibiotics were scanned to take into account the turbidity of the liposomal suspension. The concentrations of AmB and Nys in liposome preparations did not practically depend on their phospholipid, sterol, or flavonoid content and was equal to 20 ± 2 and 50 ± 3 µM, respectively. 

### 2.4. Fluorimetry of Calcein Leakage from Large Unilamellar Lipid Vesicles

**Figure 2:**
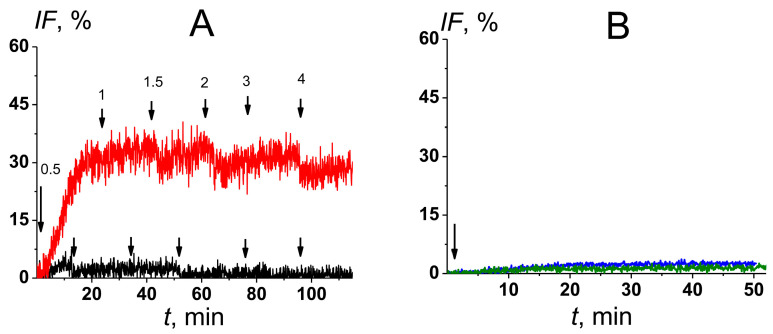
(**A**) Time dependence of calcein leakage from mammalian-like (black curve) and fungal-like lipid vesicles (red curve) at the subsequent increase in the dose of POPC/CHOL/AmB formulation in bathing solution. The moments of addition of the formulation into the liposomal suspension are indicated by the arrows. The concentrations of the monomeric AmB in µM are shown above the arrows. (**B**) Time dependence of calcein leakage from fungal-like liposomes at the addition of antibiotic-free POPC/CHOL (blue curve) and POPC/CHOL/phloretin (olive curve) formulations. The addition of the formulations until the same volume concentration as antibiotic-loaded formulations in the bathing solution was carried out at the first moment.

To monitor the membrane permeabilization induced by formulations ***1–21***, a fluorescent marker release from unilamellar lipid vesicles was performed. Liposomes mimicking the target membranes were prepared from POPC/CHOL (67/33 mol.%) (mammalian-like) and POPC/ERG (67/33 mol.%) (fungal-like) by extrusion using an Avanti Polar Lipid mini-extruder (Avanti Polar Lipids, Inc., Alabaster, AL, USA), as described earlier in [22]. Lipid chloroform stock solution was dried under a gentle stream of nitrogen, and after, it was hydrated using a 35 mM calcein solution (10 mM HEPES, pH 7.4). The suspension was subjected to six cycles of freeze-thaw, and after, it was passed through a nuclepore polycarbonate membrane (with a pore size of 100 nm) 13–15 times. Gel filtration on the sephadex G-50 column with calcein-free buffer (0.15 M NaCl, 1 mM EDTA, 10 mM HEPES, pH 7.4) was performed to remove the fluorescent marker that was not entrapped in the liposomes. The strong self-quenching of calcein at a millimolar concentration in lipid vesicles allowed us to monitor the kinetics of its fluorescence in surrounding media due to the formulation-induced marker leakage. All formulations were added to a liposome suspension up till the antibiotic concentration was 2 μM. Figure 2A shows that a further increase in the dose of the POPC/CHOL/AmB formulation in the bathing solution was not accompanied by an increase in the marker release from lipid vesicles. Figure 2B demonstrates that the effects of antibiotic-free preparations (POPC/CHOL and POPC/CHOL/phloretin), at the same volume concentration as antibiotic-loaded formulations in the bathing solution, on the permeability of fungal-like liposomes for calcein were negligible.

The calcein fluorescence induced by the addition of different formulations was measured for at least 45 min using a spectrofluorimeter, Fluorat-02-Panorama (Lumex, Saint-Petersburg, Russia), at the excitation and emission wavelengths of 490 and 520 nm, respectively. At the end of each experiment, a detergent, triton X-100, was introduced into the vesicle suspension until the concentration was 1 vol%, causing the disruption of all liposomes and producing full calcein disengagement.

Liposome permeabilization caused by the addition of the tested formulations was assessed based on the changes in the relative intensity of calcein fluorescence (*IF*, %) and calculated using the following formula:

$$IF\;=\;\;\frac{I-I_{0}}{I_{\max}/0.9-I_{0}}\cdot 100\%$$

where *I* and *I*_0_ were the intensities of calcein fluorescence after and before the introduction of the formulation, respectively, and *I*_max_ was the maximal calcein fluorescence of the sample after the addition of triton X-100 and the lysis of all liposomes. The sample dilution by triton X-100 was taken into account by correcting with a factor of 0.9.

The dependences of leakage on time *IF*(*t*) were fitted with one-exponential functions with characteristic parameters: the maximal marker leakage from mammalian-like or fungal-like lipid vesicles (*IF_CHOL_* or *IF_ERG_* respectively) and the appropriate time constant (*t*). The predicted therapeutic index (*PTI*) was calculated as a ratio of the formulation-induced mean values of *IF_ERG_* and *IF_CHOL_*.

### 2.5. Measurements of the Current-Voltage Characteristics of the Planar Lipid Bilayers Treated with Polyene Formulations

The planar lipid bilayers without solvent lenses were prepared using a method by Montal and Muller [45] on an aperture (with a diameter of 50 µm) in the Teflon film (10 µm thick) separating *cis*- and *trans*-chambers. The aperture was pretreated with hexadecane. Lipid bilayers were composed of POPC/CHOL (67/33 mol.%) (mammalian-like) and POPC/ERG (67/33 mol.%) (fungal-like) and bathed in 2.0 M KCl (5 mM HEPES, pH 7.4). After bilayer stabilization, the formulations ***1–18*** and ***21*** were introduced to the *cis*-compartment in up to 2 μM of monomeric polyene. The antibiotic-free formulations of POPC/sterol and POPC/sterol/polyphenol type were used as negative controls. To apply the transmembrane voltage (*V*) and measure the transmembrane current (*I*), Ag/AgCl electrodes with 1.5% agarose/2 M KCl bridges were used. Positive transmembrane voltage refers to a more positive potential in the *cis*-side chamber than in the *trans*-side compartment. The alteration in the formulation-induced transmembrane current was measured at the voltage ramp from −200 to +200 mV per 5 s. Membrane conductance (*G*) was determined using the ratio of the transmembrane current to the applied voltage.

Measurements of the transmembrane current were carried out using an Axopatch 200B amplifier (Molecular Devices, LLC, Orleans Drive, Sunnyvale, CA, USA) in the voltage-clamp mode. To digitize and analyze the signal, Digidata 1440A (Molecular Devices, LLC, Orleans Drive, Sunnyvale, CA, USA), pClamp 10 (Molecular Devices, LLC, Orleans Drive, Sunnyvale, CA, USA), and Origin 8.0 (OriginLab Corporation, Northampton, MA, USA) were used. Data acquisition was performed with a sampling rate of 5 kHz and 100 Hz low-pass filtering.

### 2.6. Statistical Analysis

Experiments were carried out in independent triplicates at least, and the values are presented as mean ± standard error. The Mann–Whitney–Wilcoxon’s U-test was used to compare the characteristics of membrane actions of different formulations with those of the standard preparation (***1***) (*—*p* ≤ 0.05). 

## 3. Results and Discussion

### 3.1. Modulation of Polyene-Sterol Interactions

**Figure 3:**
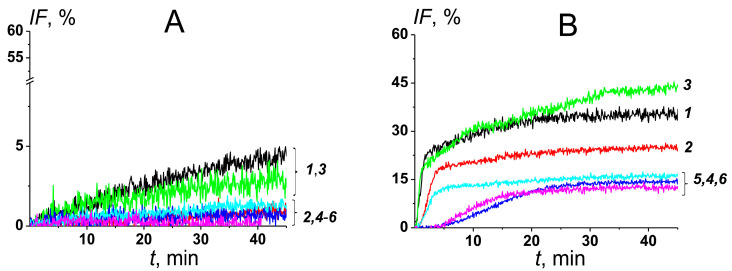
Time dependence of calcein leakage (*IF*, %) from mammalian-like (**A**) and fungal-like lipid vesicles (**B**) induced by different formulations: POPC/CHOL/AmB (***1***, black curves), POPC/DESM/AmB (***2***, red curves), POPC/7DCHOL/AmB (***3***, green curves), POPC/CAMPO/AmB (***4***, blue curves), POPC/STIGM/AmB (***5***, cyan curves), and POPC/ERG/AmB (***6***, pink curves). The addition of formulations in bathing solution up to 2 μM of monomeric AmB was carried out at the first moment.

Figure 3 demonstrates the dependence of the fluorescence marker leakage from the lipid vesicles formed by the mixture of POPC and CHOL (mimicking the mammalian membranes) (Figure 3A) or ERG (imitating fungal membranes) (Figure 3B) on the time after addition of different AmB formulations containing CHOL (***1***), DESM (***2***), 7DCHOL (***3***), CAMPO (***4***), STIGM (***5***), and ERG (***6***). The effect of the formulations ***1–6*** on the fungal-like liposomes was larger than that on mammalian-like lipid vesicles (Figure 3). Table 2 summarizes the mean values of the maximum calcein leakage from mammalian-like and fungal-like liposomes induced by the formulations ***1–6***. In the presence of formulations ***1–6***, the maximum release of the fluorescent marker from mammalian-like lipid vesicles (*IF_CHOL_*) did not exceed 5% (Figure 3A, Table 2), and the CHOL-containing formulation seemed to be the most effective. The efficacy of the formulations ***1–6*** in disengaging calcein from fungal-like liposomes (*IF_ERG_*) decreased in the following order: ***3*** ≥ ***1*** (*IF_ERG_* is about 35–40%) > ***2*** (25%) > ***4*** ≈ ***5*** (15%) > ***6*** (about 10%) (Figure 3B, Table 2). The level of calcein leakage from mammalian-like and fungal-like lipid vesicles can be used to predict the toxicity and effectiveness of the formulation, respectively. Thus, the presented results indicate that the toxicity might decrease in the series of ***1*** ≥ ***3*** ≥ ***2*** ≈ ***4*** ≈ ***5*** ≈ ***6***, while the efficiency might decrease in the series ***3*** ≥ ***1*** > ***2*** > ***5*** > ***4*** ≈ ***6***. Table 2 also shows the time constants characterizing the kinetics of calcein leakage from fungal-like liposomes induced by formulations ***1–6***. Formulations ***3***, ***4***, and ***6*** caused slower marker release than formulations ***1***, ***2***, and ***5***.

To compare the impact of different sterols, we introduced the predicted therapeutic index (*PTI*), determined as a ratio of *IF_ERG_* and *IF_CHOL_* (Table 2). *PTI* decreased in the following order: ***2*** ≈ ***3*** ≥ ***1*** ≈ ***4*** ≈ ***5*** ≥ ***6***. Thus, both formulations enriched with 7DCHOL and DESM were characterized by a promising *PTI*, but the efficacy of the DESM-containing formulations in disengaging calcein from fungal-like liposomes (*IF_ERG_*) was lower than that of the 7DCHOL-containing preparation (Table 2). Comparing the structures of the tested sterols (Figure 1E–K), we assumed that an additional double bond in the steroid core, but not in the side chain of the sterol molecule (compared to CHOL), may provide a reduction in the toxicity of the AmB lipid formulation without losing its effectiveness.

The replacement of half of the POPC content with DSPC or DPPC (with saturated acyl chains of different lengths), and negatively charged POPG, did not practically affect the ability of AmB liposomal formulation to disengage the marker from mammalian-like and fungal-like lipid vesicles compared to the standard preparation ***1***: *IF_CHOL_* was equal to 2–4% while *IF_CHOL_* was about 30% independent of phospholipid composition. These data indicate that the saturation of the phospholipid hydrocarbon tails and the length of their tails, as well as the charge of the phospholipid head, have little effect on the affinity of the polyene for the sterol and, consequently, on the toxicity and efficacy of the AmB liposomal form. Thus, varying the phospholipid component does not seem to be a promising way to improve the pharmacological properties of lipid-associated forms of polyene macrolides.

**Figure 4:**
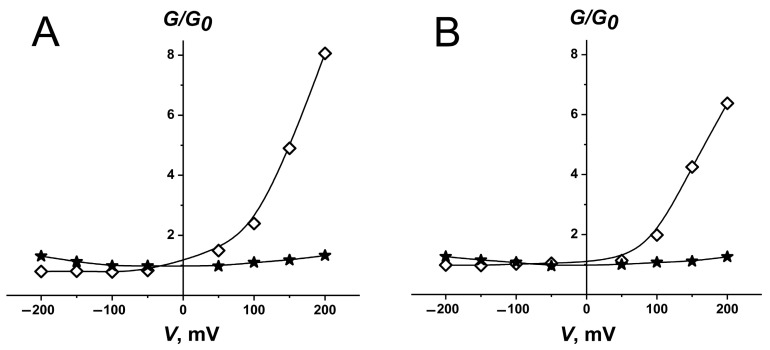
The voltage dependence of the ratio of membrane conductance produced by one-side (**◊**) and two-side (*) addition of AmB dissolved in DMSO to bilayer conductance at zero applied voltage (*G*/*G_0_*). Membranes were made from POPC/CHOL (mammalian-like) (**A**) and POPC/ERG (fungal-like) (**B**) and bathed in 2.0 M KCl (pH 7.4).

Upon the addition of the polyene antibiotic on both sides of the membrane, two half-pores, collected on opposite sides of the bilayer, associate via the formation of hydrogen bonds between the hydroxyl groups of polyene molecules and form a symmetric double-length channel [3,5,7,46]. In the case of the one-side introduction of polyene, the asymmetric half-structure forms a single-length channel localized on the side of the antibiotic addition [17,47,48]. So, the one-side addition of pure AmB into the solution bathing mammalian-like and fungal-like model membranes led to the induction of single-length AmB channels characterized by a strongly asymmetrical *G*/*G_0_*(*V*) curve (Figure 4A). Meanwhile, the two-side introduction of the nonliposomal antibiotic produced double-length AmB channels with a symmetrical *G*/*G_0_*(*V*) curve (Figure 4B). It is important to note that there is a difference in the order of magnitude in the threshold antibiotic concentrations required to produce double-length and single-length channels in favor of the latter.

**Figure 5:**
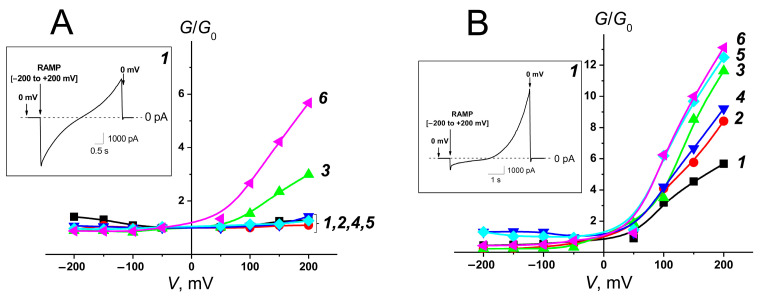
The voltage dependence of the ratio of membrane conductance produced by one-side addition of different AmB formulations to conductance at zero transmembrane voltage (*G*/*G_0_*): POPC/CHOL/AmB (***1***, black curves), POPC/DESM/AmB (***2***, red curves), POPC/7DCHOL/AmB (***3***, green curves), POPC/CAMPO/AmB (***4***, blue curves), POPC/STIGM/AmB (***5***, cyan curves), and POPC/ERG/AmB (***6***, pink curves). Mammalian-like (**A**) and fungal-like model membranes (**B**) were bathed in 2.0 M KCl (pH 7.4). *Insets*: The time dependence of the alteration in the transmembrane current induced by formulation ***1*** at the voltage ramp from −200 to +200 mV per 5 s.

The influence of liposomal AmB formulations ***1–6*** on the ion permeability of planar lipid bilayers mimicking mammalian and fungal membranes was studied. Figure 5A,B demonstrate the voltage dependences of the ratio of membrane conductance induced by the *one*-side addition of the formulations ***1–6*** into a solution bathing mammalian-like and fungal-like lipid bilayers, respectively to the membrane conductance at a transmembrane potential extrapolated to zero (*G*/*G_0_*). The *G*/*G_0_*(*V*) curves of mammalian-like lipid bilayers treated with AmB formulations ***6*** and ***3*** had an asymmetrical shape, while the *G*/*G_0_*(*V*) curves of mammalian-like model membranes modified by the formulations ***1***, ***2***, ***4***, ***5*** were symmetrical (Figure 5A). The *G*/*G_0_*(*V*) curves of fungal-like lipid bilayers were asymmetrical in the presence of all tested formulations (Figure 5B). The inserts on Figure 5A,B present examples of the time dependences of the alterations in the formulation-induced current through mammalian-like and fungal-like lipid bilayers at the voltage ramp from −200 to +200 mV per 5 s. The asymmetric and symmetric *G*-*V* curves are related to the functioning in the membranes of single-length and double-length AmB channels.

To characterize the asymmetry of the *G*-*V* curve, we calculated the ratio of the membrane conductance produced by one-side addition of the formulation at transmembrane voltage of +200 mV and −200 mV (*G_+200_*/*G_−200_*). Table 2 summarizes the values obtained for formulations enriched with different sterols. This parameter was close to 1 for mammalian-like lipid bilayers treated with formulations ***1***, ***2***, ***4***, and ***5***, and it was significantly higher for mammalian-like membranes modified by formulations ***3*** (*G_+200_*/*G_−200_* was about 7) and ***6*** (*G_+200_*/*G_−200_* was about 14). Taking into account that the formation of asymmetric channels occurs at concentrations at an order of magnitude higher than symmetric ones, the symmetry of the *G*-*V*-characteristic might be related to higher activity of AmB formulation. Thus, the data might indicate a lower toxicity of 7DHOL- and ERG-enriched AmB formulations (***3*** and ***6***) compared to formulations containing other sterols (***1***, ***2***, ***4***, and ***5***). Thus, the results of the electrophysiological assay were in good agreement with the results of calcein leakage measurements and indicated that the 7DHOL-containing preparation was the most promising in terms of reducing toxicity of AmB lipid formulations. The replacement of cholesterol in AmB-loaded formulation with any other sterol did not lead to symmetrization of the *G*-*V* characteristic of fungal-like lipid bilayers (Table 2). This meant the need to search for an additional component that can symmetrize the *G*-*V* curve of fungal-like membranes treated with the formulation. Therefore, we introduced plant polyphenols to make AmB-loaded phytosomes. 

### 3.2. Polyene-Loaded Phytosomes

According to our previously reported data, plant flavonoids, in particular phloretin, were able to increase the ability of pure AmB to form ion channels in model lipid membranes [6,7,8,43]. Biochanin A, genistein, and quercetin were shown to enhance the pore-forming activity of another polyene macrolide antibiotic, nystatin (Nys), which differs from AmB by only one double bond in the polyene chain (Figure 1L,M) [41,42].

Liposome formulations of plant flavonoids are used to overcome the low water solubility of phytochemicals [49,50,51,52,53,54]. We tried to adapt these delivery systems for polyene antibiotic administration. So, we developed innovative formulations of antibiotics that also contain flavonoids increasing the polyene pore-forming ability: polyene-loaded phytosomes.

**Figure 6:**
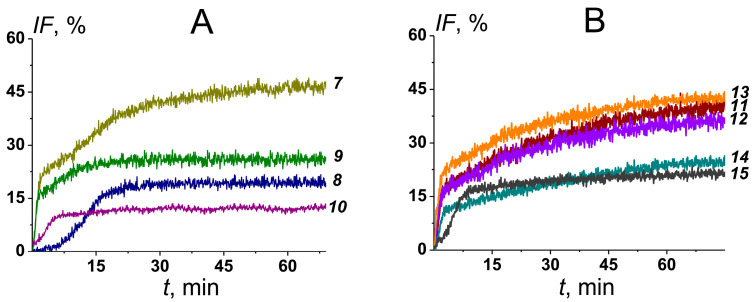
Time dependence of calcein leakage (*IF*, %) from fungal-like lipid vesicles at the addition of (**A**) AmB-loaded phytosomes containing different flavonoids, POPC/CHOL/AmB/phloretin (***7***, dark yellow curve), POPC/CHOL/AmB/biochanin A (***8***, navy curve), POPC/CHOL/AmB/genistein (***9***, olive curve), and POPC/CHOL/AmB/quercetin (***10***, purple curve); (**B**) AmB-loaded phytosomes with phloretin and composed of different sterols, POPC/DESM/AmB/phloretin (***11***, wine curve), POPC/7DCHOL/AmB/phloretin (***12***, violet curve), POPC/CAMPO/AmB/phloretin (***13***, orange curve), POPC/STIGM/AmB/phloretin (***14***, dark cyan curve), and POPC/ERG/AmB/phloretin (***15***, dark grey curve). The formulations were added in bathing solution up to 2 μM of monomeric AmB at the first moment.

Firstly, we tested the ability of AmB formulations containing POPC, CHOL, and different flavonoids, phloretin (***7***), biochanin A (***8***), genistein (***9***), and quercetin (***10***), to disengage calcein from mammalian-like and fungal-like lipid vesicles (Figure 6, Table 3). The maximum release of fluorescent marker from mammalian-like liposomes (*IF_CHOL_*) induced by AmB-loaded phytosomes ***7–10*** did not exceed 5% independently on the flavonoid type (Table 3). Figure 6A shows the time dependence of calcein leakage from fungal-like lipid vesicles after the addition of formulations ***7–10***. One can see that the efficiency expressed in *IF_ERG_*-values decreased in the following order: ***7*** (*IF_ERG_* is about 45%) > ***9*** ≈ ***8*** (20%) > ***10*** (10%). Further, we tested AmB formulations enriched with phloretin and composed of different sterols: DESM (***11***), 7DCHOL (***12***), CAMPO (***13***), STIGM (***14***), and ERG (***15***). Figure 6B demonstrates the time dependence of marker leakage from fungal-like liposomes induced by the addition of formulations ***11–15***. The efficacy of phloretin-enriched formulations containing different sterols to disengage calcein from fungal-like lipid vesicles decreased in the following order: ***7*** (45%) ≥ ***11*** ≈ ***12*** ≈ ***13*** (about 40%) > ***14*** ≈ ***15*** (25–30%) (Figure 6B, Table 3).

Table 3 also shows the time constants describing *IF*(*t*)-dependencies. Formulations containing phloretin and biochanin A produced slower marker release than formulations with genistein and quercetin and without flavonoids. The replacement of CHOL with DESM, 7DCHOL, CAMPO, and STIGM in the composition of AmB-loaded phytosomes with phloretin was accompanied by a significant slowdown in marker leakage (Table 3). 

The replacement of AmB with Nys was accompanied by a decrease in *IF_ERG_* by 15% and a dramatic slowdown in marker leakage independently on the sterol composition of phytosomes (Table 3).

**Figure 7:**
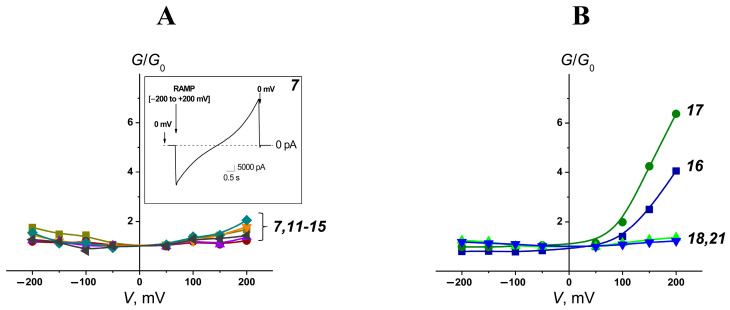
(**A**) The voltage dependence of the ratio of membrane conductance produced by one-side addition of the different phytosomes to conductance at zero transmembrane voltage (*G*/*G_0_*): POPC/CHOL/AmB/phloretin (***7***, dark yellow curve), POPC/DESM/AmB/phloretin (***11***, wine curve), POPC/7DCHOL/AmB/phloretin (***12***, violet curve), POPC/CAMPO/AmB/phloretin (***13***, orange curve), POPC/STIGM/AmB/phloretin (***14***, dark cyan curve), and POPC/ERG/AmB/phloretin (***15***, dark grey curve). *Inset*: The time dependence of the alteration in the transmembrane current induced by formulation ***7*** at the voltage ramp from −200 to +200 mV per 5 s. (**B**) The voltage dependence of the ratio of membrane conductance produced by one-side addition of different Nys formulations to conductance at zero transmembrane voltage (*G*/*G_0_*): POPC/CHOL/NyS (***16***, dark blue curve), POPC/ERG/NyS (***17***, olive curve), POPC/CHOL/NyS/phloretin (***18***, blue curve), and POPC/ERG/NyS/phloretin (***21***, green curve). Fungal-like lipid bilayers were bathed in 2.0 M KCl (pH 7.4).

The *G*/*G_0_*(*V*) curves of fungal-like membranes treated with polyene formulations containing phloretin and genistein were symmetrical, demonstrating the existence of double-length AmB channels that might relate to a decrease in the threshold concentration of the antibiotic and an increase in the efficiency of the formulation (Table 3, Figure 7A). Taking into account the *PTI* values and the magnitudes of AmB phytosome-induced currents flowing through fungal-like lipid bilayers (Table 3), POPC/CHOL/AmB/phloretin (***7***), POPC/DESM/AmB/phloretin (***11***), POPC/7DCHOL/AmB/phloretin (***12***), and POPC/ERG/AmB/phloretin (***15***) seemed to be more promising formulations. The data indicates that the introduction of phloretin into polyene liposome formulations might lead to an increase in efficiency. Similar to AmB preparations, fungal-like membranes treated with NyS-loaded phytosomes were characterized by symmetrical *G*/*G_0_*(*V*) curves, while fungal-like lipid bilayers in the presence of NyS-enriched liposomes without the flavonoid had asymmetrical *G*/*G_0_*(*V*) curves (Figure 7B). Taking into account that the inclusion of phloretin in NyS-loaded liposomes containing CHOL or ERG also led to about a 2-fold increase in *IF_ERG_*-values (*IF_ERG_* produced by POPC/CHOL/NyS (***16***) and POPC/ERG/NyS (***17***) was equal to 13-14%), we concluded that phytosomes might be considered as universal delivery systems for the administration of various polyene macrolides.

## 4. Conclusions

1. The replacement of cholesterol with its biosynthetic precursor, 7-dehydrocholesterol, led to a decrease in the ability of AmB-loaded liposomes to permeabilize lipid vesicles mimicking mammalian cell membranes. The ability of the formulation to disengage the fluorescence marker from vesicles imitating membranes of pathogenic fungi was preserved at the level of cholesterol-containing AmB lipid formulation. These facts might indicate the possibility of expanding the therapeutic window of polyene lipid formulations by varying liposome sterol content.
2. The saturation of the phospholipid hydrocarbon tails and the length of their tails, as well as the charge of the phospholipid head, had little effect on the membrane permeabilization by AmB liposomal formulations.
3. It was found that polyene-loaded phytosomes with phloretin were characterized by a greater ability to disengage calcein from lipid vesicles that mimic the membranes of fungal cells compared to liposomal formulations that did not include phloretin. Moreover, lipid bilayers mimicking fungal membranes treated with phloretin-containing polyene-loaded phytosomes demonstrated symmetrical *I*–*V* characteristics that indicated the existence of double-length polyene channels and a possible decrease in the antibiotic threshold concentration.

The results obtained using model lipid membranes that showed the possibility of improving the pharmacological properties of liposomal formulations of polyene antibiotics should be verified via a detailed study of the antifungal activity of new formulations on a wide panel of human fungal pathogens.

## Figures and Tables

**Table 1:** The composition of the tested liposome formulations.

Antibiotic-Free Liposomes	Polyene-Loaded Liposomes	Polyene-Loaded Phytosomes
POPC/CHOL	POPC/CHOL/AmB (***1***)	POPC/CHOL/AmB/phloretin (***7***)
POPC/DESM	POPC/DESM/AmB (***2***)	POPC/CHOL/AmB/biochaninA (***8***)
POPC/7DCHOL	POPC/7DCHOL/AmB (***3***)	POPC/CHOL/AmB/genistein (***9***)
POPC/CAMPO	POPC/CAMPO/AmB (***4***)	POPC/CHOL/AmB/quercetin (***10***)
POPC/STIGM	POPC/STIGM/AmB (***5***)	POPC/DESM/AmB/phloretin (***11***)
POPC/ERG	POPC/ERG/AmB (***6***)	POPC/7DCHOL/AmB/phloretin (***12***)
POPC/CHOL/phloretin	POPC/POPG/CHOL/AmB	POPC/CAMPO/AmB/phloretin (***13***)
POPC/CHOL/biochanin A	POPC/DPPC/CHOL/AmB	POPC/STIGM/AmB/phloretin (***14***)
POPC/CHOL/genistein	POPC/DSPC/CHOL/AmB	POPC/ERG/AmB/phloretin (***15***)
POPC/CHOL/quercetin	POPC/CHOL/NyS (***16***)	POPC/CHOL/NyS/phloretin (***18***)
POPC/DESM/phloretin	POPC/ERG/NyS (***17***)	POPC/DESM/NyS/phloretin (***19***)
POPC/7DCHOL/phloretin		POPC/7DCHOL/NyS/phloretin (***20***)
POPC/CAMPO/phloretin		POPC/ERG/NyS/phloretin (***21***)
POPC/STIGM/phloretin		
POPC/ERG/phloretin		

**Table 2:** The characteristics of the membrane activity of AmB formulations with different sterols.

Formulation	Mammalian-like Membranes		Fungal-like Membranes			*PTI*
	*IF_CHOL_, %*	*G_+200_*/*G_−200_*	*IF_ERG_, %*	*t*, min	*G_+200_*/*G_−200_*	
POPC/CHOL/AmB (***1***)	4 ± 1	1.0 ± 0.1	34 ± 3	6 ± 1	13 ± 1	9 ± 3
POPC/DESM/AmB (***2***)	2 ± 1 *	1.0 ± 0.1	25 ± 2 *	4 ± 1 *	37 ± 2 *	13 ± 7
POPC/7DCHOL/AmB (***3***)	3 ± 1 *	7 ± 1 *	37 ± 7	18 ± 2 *	49 ± 3 *	12 ± 6
POPC/CAMPO/AmB (***4***)	2 ± 1 *	1.3 ± 0.2	14 ± 3 *	14 ± 1 *	11 ± 2	7 ± 5
POPC/STIGM/AmB (***5***)	2 ± 1 *	1.2 ± 0.3	15 ± 1 *	5 ± 1	10 ± 2	8 ± 4
POPC/ERG/AmB (***6***)	2 ± 1 *	14 ± 1 *	9 ± 3 *	18 ± 1 *	30 ± 3 *	5 ± 4

*IF_CHOL_*, *IF_ERG_*—formulation-induced maximal leakage of calcein from unilamellar mammalian-like and fungal-like lipid vesicles, respectively; *G_+200_*/*G_−200_*—the ratio of membrane conductance produced by one-side addition of the formulations at the transmembrane voltage of +200 mV to conductance at −200 mV; *t*—the characteristic parameter of the time dependences of marker leakage from fungal-like liposomes; *PTI*—predicted therapeutic index. *—*p* ≤ 0.05 (Mann–Whitney–Wilcoxon’s test vs. standard formulation ***1***).

**Table 3:** The characteristic parameters of the membrane activity of developed polyene-loaded phytosomes.

Formulation	*IF_CHOL_, %*	*IF_ERG_, %*	*t*, min	*PTI*	Fungal-like Membranes	
					*G_+200_*/*G_−200_*	*I*_+200_, μA
POPC/CHOL/AmB/phloretin (***7***)	4 ± 1	45 ± 5 *	15 ± 2 *	11 ± 4	1.1 ± 0.1 *	30 ± 3 *
POPC/CHOL/AmB/biochaninA (***8***)	3 ± 1	20 ± 2 *	14 ± 2 *	7 ± 3	4 ± 1 *	–
POPC/CHOL/AmB/genistein (***9***)	2 ± 1 *	23 ± 3 *	5 ± 1	12 ± 3	1.0 ± 0.1 *	–
POPC/CHOL/AmB/quercetin (***10***)	2 ± 1 *	10 ± 2 *	6 ± 1	5 ± 4	60 ± 7 *	–
POPC/DESM/AmB/phloretin (***11***)	3 ± 1 *	42 ± 6 *	27 ± 2 *	14 ± 7	1.1 ± 0.1 *	25 ± 6 *
POPC/7DCHOL/AmB/phloretin (***12***)	4 ± 1	38 ± 2	23 ± 3 *	10 ± 3	1.1 ± 0.1 *	24 ± 2 *
POPC/CAMPO/AmB/phloretin (***13***)	5 ± 1	38 ± 3	21 ± 2 *	8 ± 2	1.1 ± 0.1 *	29 ± 8 *
POPC/STIGM/AmB/phloretin (***14***)	3 ± 1 *	27 ± 4	33 ± 4 *	9 ± 4	1.3 ± 0.1 *	10 ± 2 *
POPC/ERG/AmB/phloretin (***15***)	2 ± 1 *	26 ± 3 *	18 ± 3 *	13 ± 8	1.0 ± 0.1 *	27 ± 6 *
POPC/CHOL/NyS/phloretin (***18***)	2 ± 1 *	30 ± 6	36 ± 1 *	15 ± 10	1.1 ± 0.1 *	8 ± 2
POPC/DESM/NyS/phloretin (***19***)	2 ± 1 *	27 ± 5	35 ± 2 *	14 ± 9	–	–
POPC/7DCHOL/NyS/phloretin (***20***)	2 ± 1 *	30 ± 4	36 ± 2 *	15 ± 10	–	–
POPC/ERG/NyS/phloretin (***21***)	2 ± 1 *	30 ± 5	35 ± 1 *	15 ± 10	1.0 ± 0.1 *	12 ± 2 *

*IF_CHOL_, IF_ERG_*—formulation-induced maximal leakage of calcein from unilamellar mammalian-like and fungal-like lipid vesicles, respectively; *G_+200_*/*G_−200_*—the ratio of membrane conductance produced by one-side addition of the formulations at a transmembrane voltage of +200 mV to conductance at −200 mV; *t*—the characteristic parameter of the time dependences of marker leakage from fungal-like liposomes; *PTI*—predicted therapeutic index; *I*_+200—_the current flowing through fungal-like membrane produced by one-side addition of the formulation at transmembrane potential of +200 mV (*I*_+200_ induced by formulation ***1*** was equal to 3 ± 1 μA). *—*p* ≤ 0.05 (Mann–Whitney–Wilcoxon’s test vs. standard formulation ***1***).

## Data Availability

The data presented in this study are available in this article.
